# Single-cell sequencing reveals novel proliferative cell type: a key player in renal cell carcinoma prognosis and therapeutic response

**DOI:** 10.1007/s10238-024-01424-x

**Published:** 2024-07-25

**Authors:** Bicheng Ye, Hongsheng Ji, Meng Zhu, Anbang Wang, Jingsong Tang, Yong Liang, Qing Zhang

**Affiliations:** 1 School of Clinical Medicine, Yangzhou Polytechnic College, Yangzhou, China; 2 Department of Urology, Changhai Hospital, Naval Medical University (Second Military Medical University), Shanghai, China; 3 Department of Urology, Lianshui People’s Hospital of Kangda College Affiliated to Nanjing Medical University, Huai’an, China; 4 Department of Geriatrics, The Affiliated Huaian Hospital of Xuzhou Medical University, Huaian Second People’s Hospital, Huaian, China; 5 Department of General Surgery, Northern Jiangsu People’s Hospital Affiliated to Yangzhou University, Yangzhou, China; 6 Department of Medical Laboratory, Huai’an Second People’s Hospital Affiliated to Xuzhou Medical Universit, Huaian, China; 7 Department of Hepatology, Huai’an No. 4 People’s Hospital, Huai’an, China

**Keywords:** Renal cell carcinoma, Artificial intelligence, Single-cell RNA sequencing, Immunotherapy, Prognosis

## Abstract

Renal cell carcinoma (RCC) is characterized by a variety of subtypes, each defined by unique genetic and morphological features. This study utilizes single-cell RNA sequencing to explore the molecular heterogeneity of RCC. A highly proliferative cell subset, termed as “Prol,” was discovered within RCC tumors, and its increased presence was linked to poorer patient outcomes. An artificial intelligence network, encompassing traditional regression, machine learning, and deep learning algorithms, was employed to develop a Prol signature capable of predicting prognosis. The signature demonstrated superior performance in predicting RCC prognosis compared to other signatures and exhibited pan-cancer prognostic capabilities. RCC patients with high Prol signature scores exhibited resistance to targeted therapies and immunotherapies. Furthermore, the key gene CEP55 from the Prol signature was validated by both proteinomics and quantitative real time polymerase chain reaction. Our findings may provide new insights into the molecular and cellular mechanisms of RCC and facilitate the development of novel biomarkers and therapeutic targets.

## Introduction

As the most common form of kidney cancer, renal cell carcinoma (RCC) accounts for approximately 85% of reported renal malignancies [1]. At least, 12 distinct subtypes of RCC have been identified, each characterized by different morphological features and genetic abnormalities specific to that subtype [2]. The 3 major subtypes, clear cell RCC, papillary RCC, and chromophobe RCC, together comprise over 90% of diagnosed RCC cases [3]. RCC has a complex, multifactorial pathology involving unhealthy lifestyle factors, genetic mutations, and cellular damage [1]. Similar to most malignancies, the molecular heterogeneity characteristic of RCC impedes effective therapeutic interventions [4, 5]. Moreover, the 5-year overall survival (OS) rate for patients with metastatic RCC is notably below 10% [6–8]. As such, delving into the intricate molecular and cellular attributes of RCC could offer critical insights beneficial for potential biomarker identification and treatment modalities.

Single-cell RNA sequencing (scRNA-seq) is an advanced and novel transcriptomic technology that facilitates the profiling of gene expression at an individual cellular resolution [9]. Exploiting the high-throughput capabilities of scRNA-seq enables researchers to comprehensively delineate the diverse tumor-resident cell populations, which may otherwise be masked by the bulk profiles attained through conventional transcriptomic methodologies [10]. With ongoing refinements in scRNA-seq protocols, the application of this technology to analyze solid malignancies, including RCC, has become progressively more ubiquitous [11, 12]. However, detecting rare cellular subsets remain challenging in scRNA-seq. Thus, integrating multiple datasets are crucial to characterize these scarce populations.

Machine learning and deep learning serve as potent instruments for tackling intricate medical issues through the utilization of extensive clinical data sets [13]. These methodologies have consistently demonstrated their prowess and efficiency in prediction and clustering tasks [14]. By employing these advanced technologies, we can delve into the mechanisms of therapy resistance across various levels, such as transcriptional, epigenetic, and translational, uncovering more insights to enhance treatment efficacy [15–17]. Consequently, we have developed a novel artificial intelligence network that integrates traditional regression algorithms, machine learning, and deep learning, encompassing a total of 12 algorithms and 184 algorithm combinations, significantly surpassing the previous 101 algorithm combinations [18]. This exhaustive strategy enables us to more precisely analyze and predict clinical outcomes in RCC patients.

We hypothesized that consolidating scRNA-seq data from multiple cohorts may elucidate presently unrecognized cellular subsets associated with RCC that influence patient prognosis as their representation increases within the malignant tissue. In this study, we delineated and profiled a highly proliferative RCC cellular subset designated “Prol.” We demonstrated that expanded representation of Prol cells within tumors correlated with inferior patient prognosis. Additionally, we effectively implemented a novel artificial intelligence (AI) network to develop a signature derived from Prol cells marker genes capable of precisely determining Prol cell abundance and forecasting clinical outcomes in RCC patients. Our findings may uncover novel facets of RCC pathobiology, holding promise for enabling enhanced patient subclassification and targeted therapeutics for individuals with this malignancy.

## Methods

### Collection and preprocessing of scRNA RNA-seq data

scRNA-seq data from two cohorts containing primary RCC tumors and non-tumor kidney tissue were downloaded [11, 12], comprising 11 samples from primary RCC tumors and 8 samples from adjacent normal kidney tissue. We applied filters to the gene-cell matrix to eliminate cells with low quality, with the quality control standards referring to previous research [11, 12]. A total of 77,817 cells were analyzed using the Seurat (version 4.2.1) R package [19]. Gene expression levels were normalized using the “LogNormalize” method with a scale factor of 10,000 in Seurat. Highly variable genes were identified (*n* = 2000), and their expression values were scaled prior to conducting principal component analysis (PCA). Batch effects were regressed out using the Harmony (version 0.1.1) R package [20]. Data analysis was performed using functions from the Harmony and Seurat R packages, including NormalizeData, FindVariableFeatures, ScaleData, RunPCA, FindNeighbors, FindClusters, and RunUMAP. Cell cycle phase scoring was done with the CellCycleScoring function in Seurat [21]. Distinct marker genes were utilized to sort the main cell types. Epithelial cells were annotated with: KRT19, KRT18, KRT8, and EPCAM. T/NK cells were characterized by: PTPRC, CD3D, CD3E, and NKG7. B cells were identified with: IGHG1, JCHAIN, and CD79A. Myeloid cells were marked by: CD68, CD14, CD163, CD1C, CLEC4C, and KIT. Endothelial cells were characterized by: VWF and PECAM1, and fibroblasts were denoted with ACTA2, COL1A1, and COL1A2.

### Collection and preprocessing of bulk RNA-seq data

Transcriptomic and clinical data of RCC patients were obtained from The Cancer Genome Atlas (TCGA), Gene Expression Omnibus (GEO), and ArrayExpress databases. A total of 1075 samples across four datasets were analyzed, comprising 879 RCC patients from the TCGA dataset [22] (including TCGA-KICH, TCGA-KIRC, and TCGA-KIRP cohorts), 40 patients from the GSE22541 [23], 55 patients from the GSE167573 [24], and 101 patients from the E-MTAB-1980 [25]. Moreover, we downloaded the data from three distinct RCC cohorts that are undergoing varying treatment regimens, namely sunitinib, everolimus, nivolumab, or a combination of atezolizumab and bevacizumab. Of these, the E-MTAB-3267 cohort encompasses 53 RCC patients undergoing sunitinib treatment [26]. The Cancercell cohort, on the other hand, contains a broader spectrum with 416 RCC patients being treated with sunitinib, along with another set of 407 RCC patients on a combination therapy of atezolizumab and bevacizumab [27]. The CheckMate cohort builds up with 130 RCC patients treated with everolimus, alongside an additional 181 RCC patients under a therapeutic regimen of nivolumab [28]. Notably, their transcriptomic data were all derived prior to the initiation of their respective treatments.

For the TCGA and GSE167573 cohorts, raw read counts were normalized to transcripts per kilobase million (TPM) values. The processed expression data accessed from the other cohorts were directly downloaded through their respective data portals. Gene expression data across all cohorts were log2 transformed, z-score normalized, and batch effects were removed using the surrogate variable analysis (SVA) algorithm [29].

### Abundance of cell types in bulk RNA-seq data

Cell type abundances were estimated from the bulk kidney expression data in the TCGA cohort using the BisqueRNA R package (version 1.0.5) [30]. A PCA-based approach was utilized to deconvolute the 7 main kidney cell types based on scRNA data for downstream analyses.

### Associations between patient survival and estimated cell type proportions

Associations between estimated cell type proportions and prognosis were assessed using Cox proportional hazards regression in the TCGA-RCC cohort. Patients were stratified into high and low Prol cell abundance groups based on optimal cutoffs determined by the survminer R package (version 0.4.9). Kaplan–Meier analysis with the log-rank test was utilized to compare survival differences between the two groups using the survival R package (version 3.4–0).

### SCENIC analysis

The task of analyzing the transcription factors (TFs) and distinguishing stable cell states was executed using the SCENIC package in R (version 1.2) [31]. We excluded genes that showed low expression levels (UMI counts < 3 in more than 1% of the cells) along with genes observed in less than 1% of the cellular population.

### Development of signatures using an artificial intelligence network

We aimed to develop an accurate and stable Prol signature for predicting the prognosis of RCC patients. We constructed an AI network based on 184 algorithm combinations, integrating 12 algorithms from traditional regression, machine learning and deep learning. These algorithms included stepwise Cox, random survival forest (RSF), gradient boosting machine (GBM), supervised principal components (SuperPC), least absolute shrinkage and selection operator (LASSO), survival support vector machine (survival-SVM), Ridge, elastic network (Enet), deephit survival neural network (DeepHit), deepsurv survival neural network (DeepSurv), Coxboost, and variable selection oriented LASSO bagging algorithm (VSOLassoBag). Initially, univariate Cox regression was performed to identify prognostic Prol cell markers in the TCGA cohort at *P* < 1e-6 significance. Subsequently, 184 algorithm combinations were applied to these markers to develop predictive models, still within the TCGA cohort. The predictive performance of each algorithm combination was then evaluated using C-indices across all validation cohorts. The optimal combination was selected as that with the maximum mean C-index. For the purpose of pinpointing the most important genes and establishing a robust Prol signature, we employed the VSOLassoBag package of R (version 0.99.1), applying bootN at 10, a.family set to cox, and bagFreq.sigMethod labeled as “CEP.” Following this, we initiated the Random Survival Forest (RSF) algorithm, making use of the RandomForestSRC package (version 3.1.1) on R.

### Analysis of the expression and prognostic value of CEP55 at the protein level

The Clinical Proteomic Tumor Analysis Consortium (CPTAC) (https://proteomics.cancer.gov/programs/cptac), initiated in 2011, seeks to broaden our understanding of cancer through the lens of proteome and proteogenomics [32]. In our present study, we harnessed the power of the CPTAC dataset to delve into the expression levels of CEP55 protein within tumor and normal tissues. Additionally, we probed the prognostic implications of CEP55 at the protein level.

### qRT-PCR

To gauge the expression of CEP55 in both tumor and adjacent renal tissue samples, we employed quantitative real time polymerase chain reaction (qRT-PCR), with the analyzed renal tissue samples comprising 22 ccRCC tissues sourced from Changhai Hospital. The choice of primer sequences for the qRT-PCR included a forward primer: AGTAAGTGGGATCGAAGCCT and a reverse primer: CTCAAGGACTCGAATTTTCTCCA. Our previous study [16] provides a reference to the primer sequences adopted for *β*-actin.

## Statistical analysis

Numerical data were put through the Wilcoxon test. The formulation of survival curves was made possible through the utilization of Survminer and Survival packages in R. We conducted both univariate and multivariate Cox regression analyses to gauge the clinical factor independence of the Prol signature. To ascertain predictive sensitivity and specificity for survival or response, we deployed Receiver Operator Characteristic curve (ROC) analysis. Unless specified differently, we defined statistical significance as a P value that fell below 0.05. The R software, version 4.2.3, was instrumental in facilitating all the analyses.

## Results

### Unveiling a cell cycle-related cell type

**Fig. 1:**
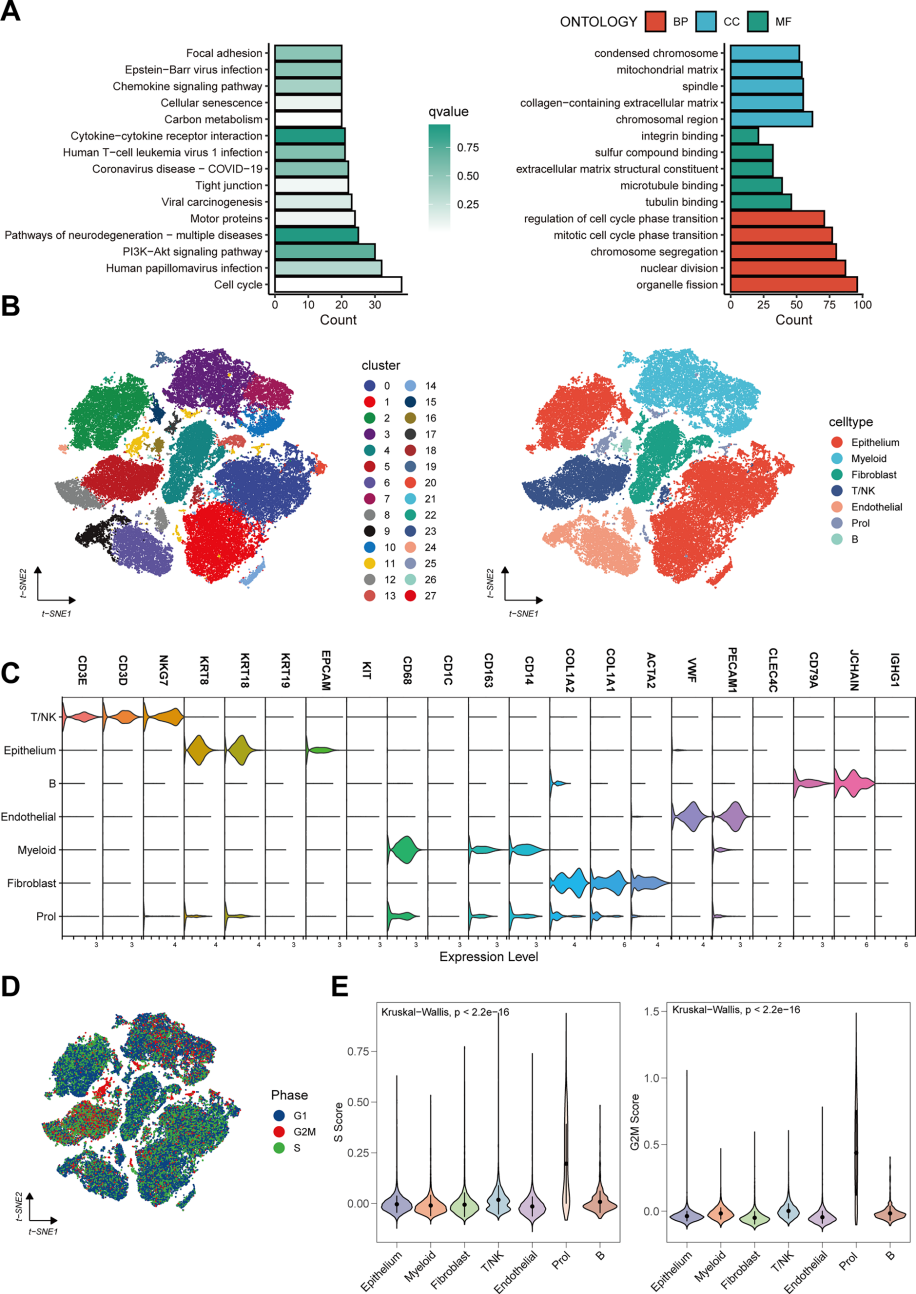
Unveiling and profiling a proliferative (Prol) cell type. **A** KEGG (left) and GO (right) enrichment analysis of prognosis-related genes in renal cell carcinoma (RCC). **B** The visualization of 66,817 cells using Uniform Manifold Approximation and Projection (UMAP) revealed the integration of datasets to remove the batch effect. Clusters were categorized into (left) 28 subtypes and (right) 7 major cell types. **C** Distinct marker genes of 7 main cell types. **D** Coloring of cells based on the inferred cell cycle phase from single-cell RNA sequencing (scRNA-seq) data. **E** Significantly higher S phase (left) and G2M phase (right) scores in Prol cells

In the TCGA-RCC cohort, we initiated our study by uncovering genes linked to OS via univariate Cox regression (*P* < 1e-6), discovering that these genes were concentrated in cell cycle-associated pathways (Fig. 1A). This prompted the assumption of a unique cell type within the RCC that is distinguished by its cell cycle characteristics. Incorporating clustering of scRNA-seq data, we established 28 distinct cell types altogether, subsequently merged and partitioned into seven prime cell types (Fig. 1B). Distinct marker genes were utilized to sort the main cell types (Fig. 1C). Intriguingly, a novel grouping, which we defined as Proliferative (Prol) cell type, was unveiled with the G2M phase being the predominant stage for most cells within this category (Fig. 1D). Prol cells notably demonstrated elevated scores for S and G2M phases when juxtaposed with other cells (Fig. 1E).

### The upregulated abundance of Prol cells correlates with unfavorable prognosis

**Fig. 2:**
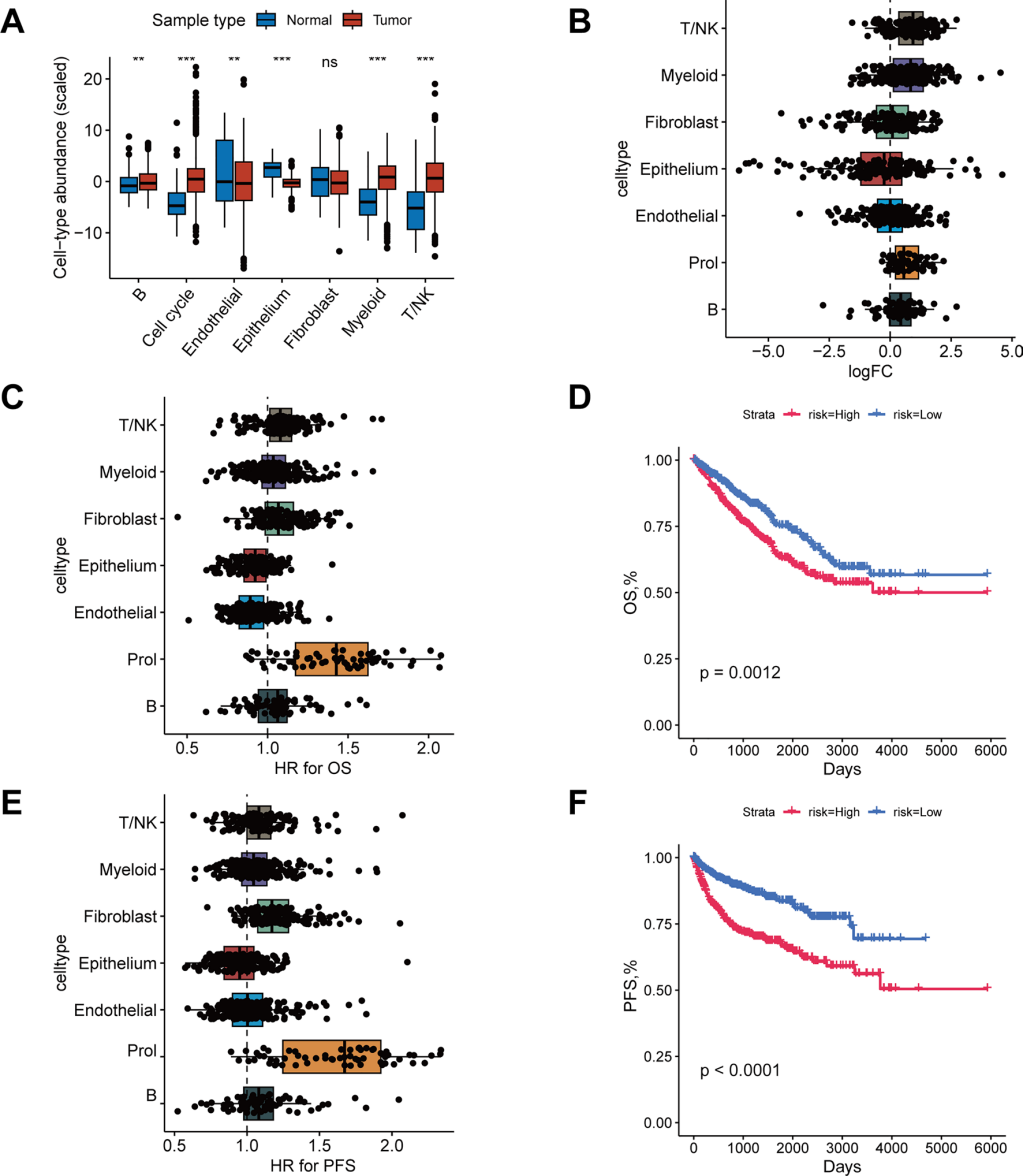
Higher Prol cell type abundance in RCC, linked with poor prognosis. **A** Estimation of major cell types proportions from scRNA data in RCC bulk RNA sequencing (RNA-seq), with differential testing between tumor and non-tumor samples. **B** Log2 fold-changes (Log2FC) of cell type marker genes between tumor and adjacent non-tumor samples. Each plotted dot represents a gene, with its log2FC on the x-axis and its cell type on the y-axis. **C** Prol cell type’s association with poor overall survival (OS), manifested through HR values for cell type marker genes from Cox proportional hazards regression. Each dot is a gene, with HR value on x-axis and cell type on y-axis. **D** Kaplan–Meier curves of OS according to the Prol cells abundance. **E** Prol cell type’s association with poor progression-free survival (PFS), manifested through HR values for cell type marker genes from Cox proportional hazards regression. Each dot is a gene, with HR value on x-axis and cell type on y-axis. **F** Kaplan–Meier curves of PFS according to the Prol cells abundance

In order to confirm the cell type composition changes observed in our scRNA-seq data within the broader context of RCC, we employed bulk RNA-seq data from the TCGA for the estimation of the seven main cell type abundances. Notably, the Prol cell abundance in RCC samples showed a statistically significant rise (*P* < 0.001) in comparison with non-tumor samples (Fig. 2A). Prol marker genes identified from the scRNA data with a log2 fold change (FC) > 1 were predominantly highly expressed in the bulk RNA-seq data (Fig. 2B). Prol marker genes demonstrated the highest hazard ratio for both OS and progression-free survival (PFS) relative to all other cell types (Fig. 2C, E). Along the same lines, the Kaplan–Meier curve revealed that an elevated abundance of Prol cells correlated with a worse OS and PFS (*P* < 0.05) (Fig. 2D, F). These findings suggest that Prol cells play a crucial role in determining the prognosis of RCC patients.

### Unique TF profile associated with Prol cells

**Fig. 3:**
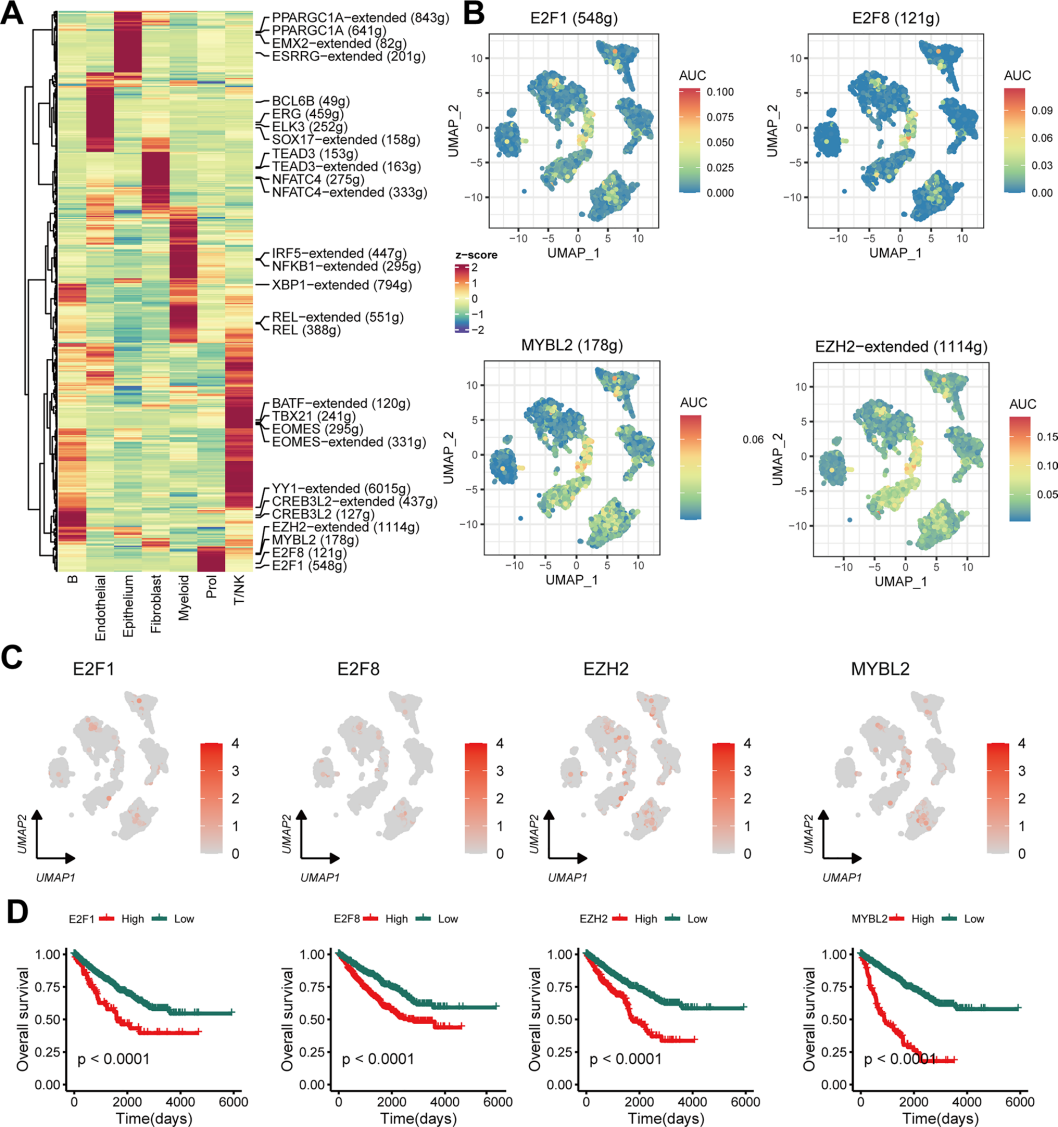
Unique transcription factor (TF) activity associated with Prol cells. **A** Heatmap showing differences in TF activity scored by SCENIC. **B** TF activity of E2F1, E2F8, MYBL2 and EZH2 projected on UMAP. **C** TF expression of E2F1, E2F8, MYBL2 and EZH2 projected on UMAP. **D** Kaplan–Meier survival curves for E2F1, E2F8, MYBL2 and EZH2

Prior investigations [33] have indicated the fundamental role that TFs play in cell fate determination. Distinct patterns of these TFs were exclusive to each cell type, as displayed in Fig. 3A. We observed a significant enrichment of E2F1, E2F8, MYBL2 and EZH2 regulons activity in the Prol cells (Fig. 3B). The expression levels of these four TFs, as well as their correlation with lower survival rates in RCC patients from the TCGA cohort, are displayed in Fig. 3C, D (*P* < 0.05), respectively. The four TFs have been shown to be associated with the cell cycle [34–36]. This suggests that these four TFs may play a crucial role in maintaining the ‘proliferative’ characteristics of RCC.

### Construction of the Prol signature

**Fig. 4:**
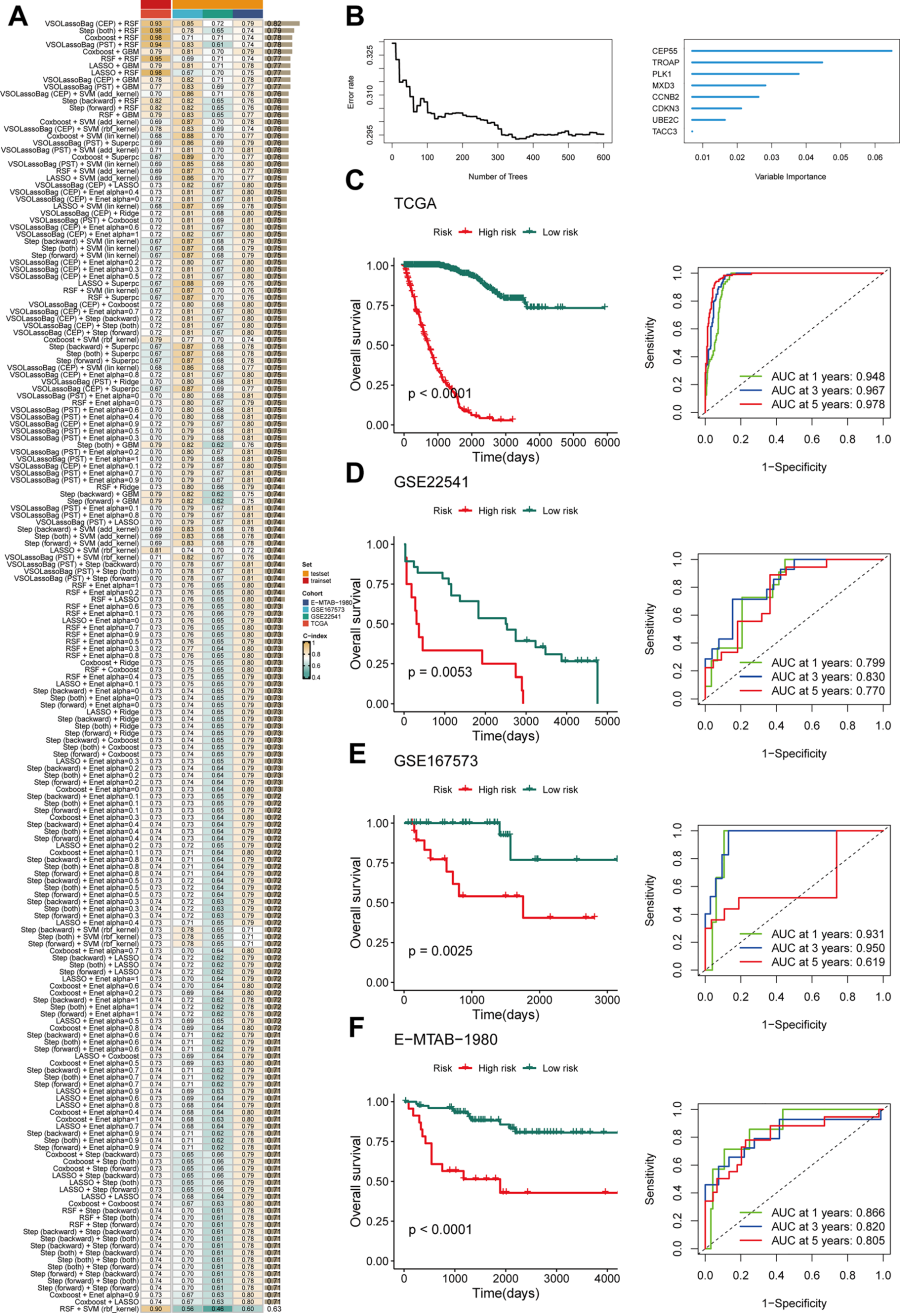
Development and validation of an artificial intelligence (AI) network using 184 algorithm combinations. **A** Evaluation and C-index computation for 184 prediction models across all datasets. **B** Determination of the number of trees by minimizing error. **C** Variable importance of the top 8 genes determined using the random survival forest (RSF) algorithm. **C**–**F** Kaplan–Meier survival analysis (left) and receiver operating characteristic (ROC) (right) curves for OS in the TCGA, GSE22541, GSE167573 and E-MATB-1980 cohorts

To enhance prognostic prediction in RCC and quantify the abundance of Prol cells by employing key genes, we developed a Prol signature based on the AI network. We first performed univariate Cox analysis to select the most specific Prol cell markers (log2 fold change > 0.5, adjusted *P*-value = 0) with prognostic value (*P* < 1 * 10^-6). Consequently, 184 algorithm combinations on the TCGA group were tested, and the C-index was calculated for all on the verifying groups. The highest mean C-index was registered at 0.764 by the combination of VSOLassoBag and RSF (Fig. 4A). VSOLassoBag identified 8 genes (Fig. 4B) employed by RSF in building the Prol signature. Stratification of RCC patients into high and low-risk categories was conducted based on the optimum cutoff. The high-risk group mirrored a significantly inferior overall survival (OS) compared to the low-risk group across all sets (*P* < 0.05) (Fig. 4C–F). Furthermore, ROC curves reflected the solid and dependable performance of the Prol signature across all sets (Fig. 4C–F). Thus, the Prol signature shows potential as a predictor of outcomes in RCC.

### Comparison of prognostic signatures

**Fig. 5:**
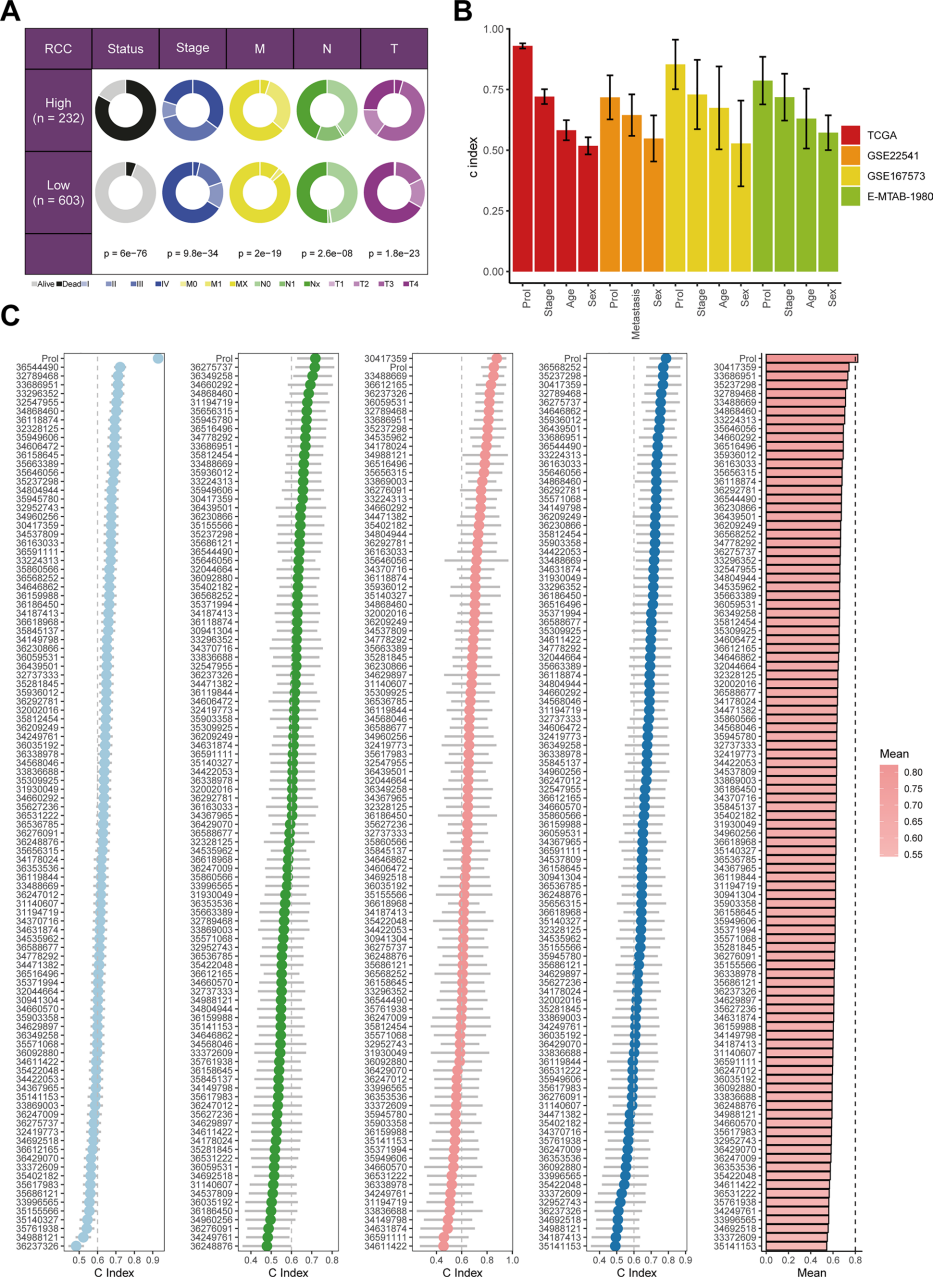
Comparison between the Prol signature and other models. **A** Circos plot of different clinical factors in two Prol signature score groups in the TCGA cohort. **B** The C-index of the Prol signature and other clinical factors in the TCGA, GSE22541, GSE167573 and E-MATB-1980 cohorts. **C** The C-index of the Prol signature and other models developed in the TCGA, GSE22541, GSE167573 and E-MATB-1980 cohorts. The numbers in the graph are PMID

We found that the Prol signature was significantly positively correlated with survival status, tumor stage, as well as TNM stage was evaluated within the TCGA dataset (Fig. 5A). Based on the C-index across all datasets, the Prol signature surpassed age, gender, tumor stage, and TNM stage (Fig. 5B). Furthermore, the Prol signature was contrasted with 101 alternate signatures within the TCGA, GSE22541, GSE167573, and E-MTAB-3892 cohorts (Fig. 5C). The Prol signature attained the supreme C-index in all mentioned cohorts among all assessed signatures (Fig. 5C). This accentuates our signature’s potential to function as a reliable substitute for forecasting prognosis among patients with RCC.

### Predictive value of targeted therapy and immunotherapy benefits

**Fig. 6:**
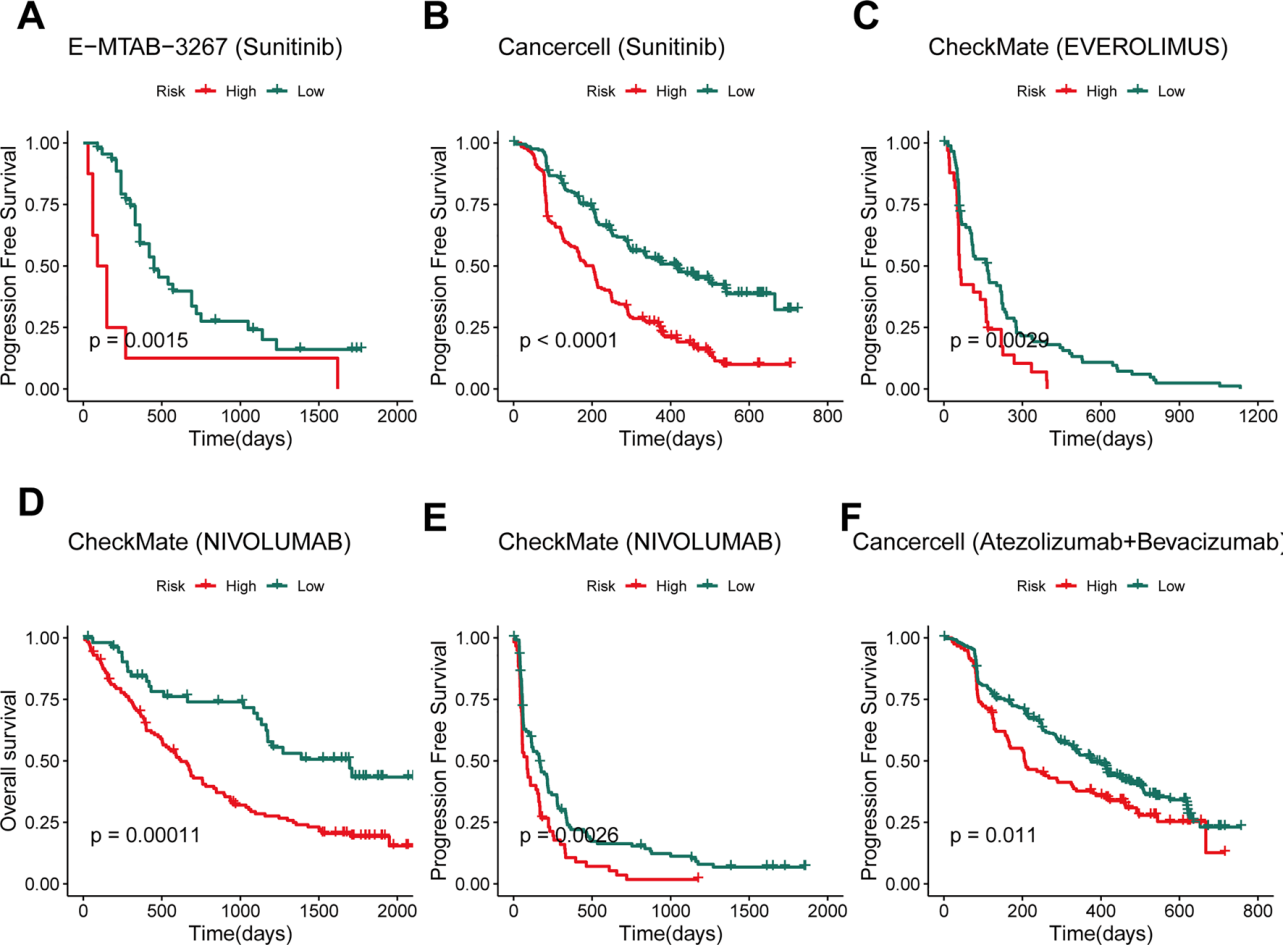
Predictive value of targeted therapy and immunotherapy benefits. **A–B** Kaplan–Meier survival analysis for PFS in the sunitinib treatment cohorts. **C** Kaplan–Meier survival analysis for PFS in the everolimus treatment cohort. **D**–**E** Kaplan–Meier survival analysis for OS and PFS in the nivolumab treatment cohorts. **F.** Kaplan–Meier survival analysis for PFS in the atezolizumab combined with bevacizumab treatment cohort

To assess whether Prol signature could predict the outcome of targeted therapy and immunotherapy, we performed KM curves in the relevant cohort. We found that patients in the high-risk group had a worse prognosis compared to those in the low-risk group, regardless of treatment with sunitinib (Fig. 6A, B) or everolimus (Fig. 6C) or nivolumab (Fig. 6D, E) or atezolizumab combined with bevacizumab (Fig. 6F) (all *P* < 0.05). Taken together, patients with low Prol signature score tend to be sensitive to sunitinib or everolimus or nivolumab or atezolizumab combined with bevacizumab.

### Universal application of the Prol signature across all cancers

**Fig. 7:**
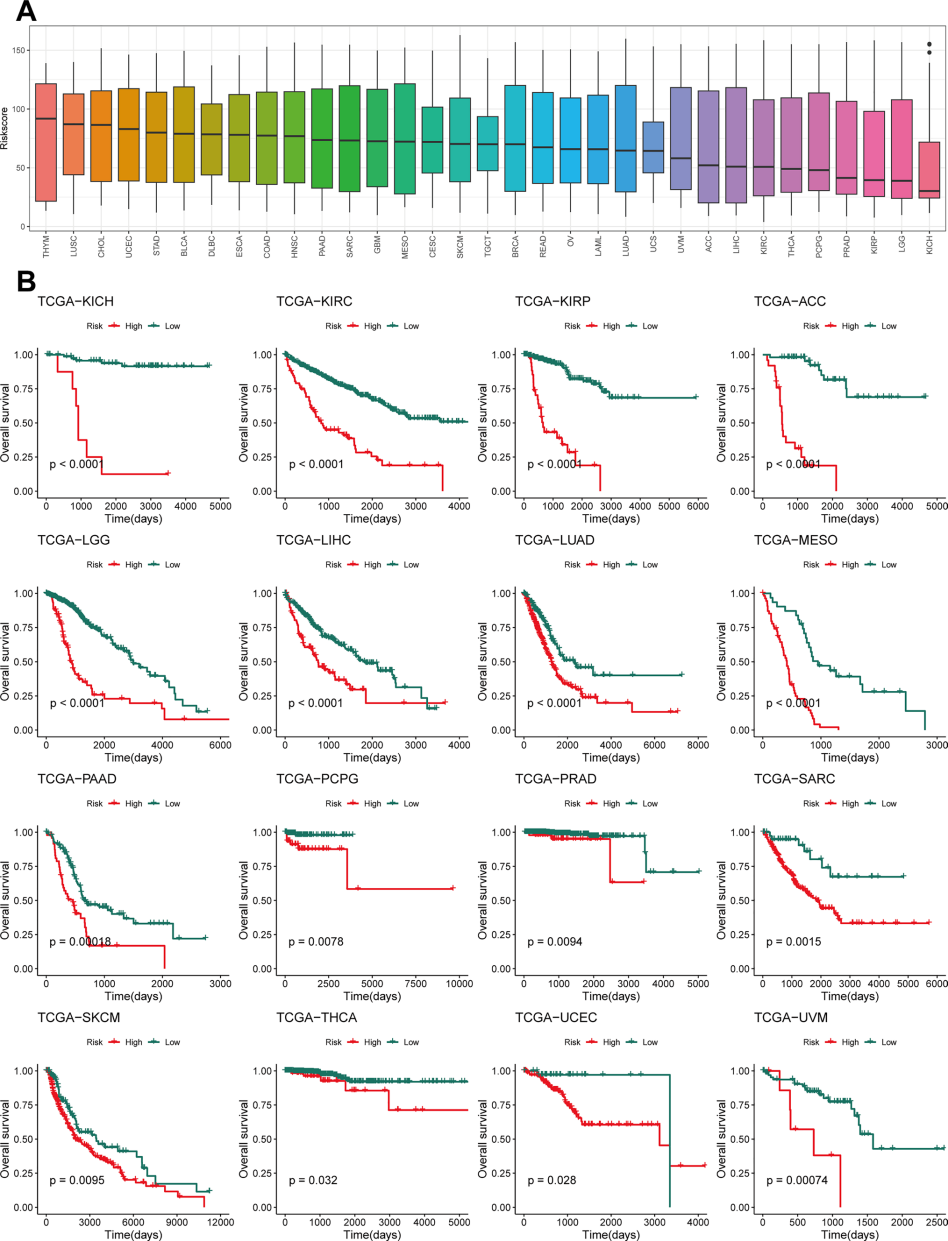
Landscape of Prol signature across various cancer types. **A** Prol signature score for 33 cancers. **B** Kaplan–Meier curves of OS according to the Prol signature

The Prol signature score for 33 unique cancers was estimated from the TCGA (Fig. 7A), which were segmented into high-risk and low-risk categories. A direct correlation was found between escalated Prol signature scores and deteriorating prognosis across every one of the 16 cancers (Fig. 7B). Such insights emphasize the paramount role of Prol cells in determining the patient’s outcome.

### Validation of expression and prognosis of markers from the Prol signature

**Fig. 8:**
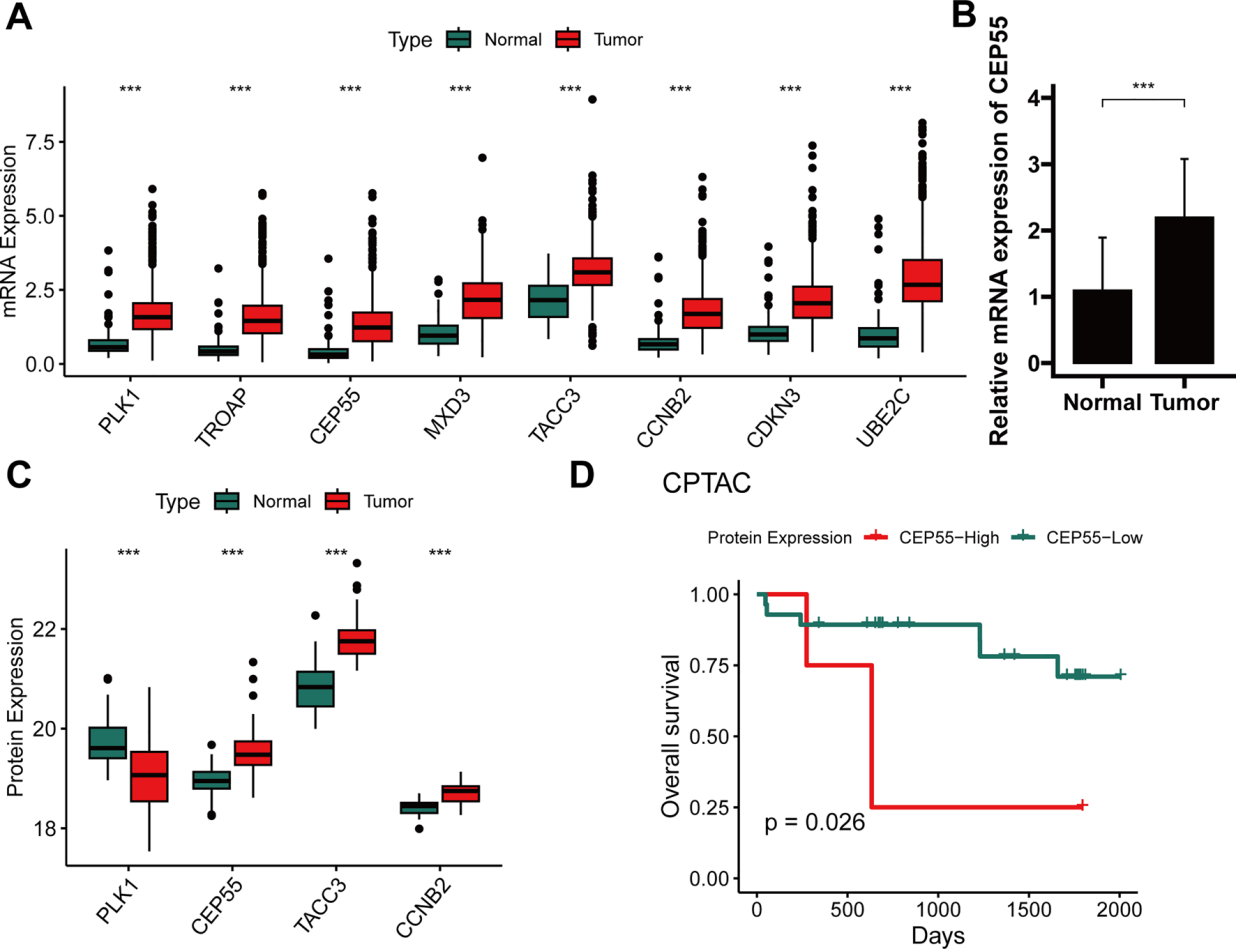
Validation of expression of genes from the Prol signature. **A** Differential mRNA expression of 8 model genes in normal and RCC samples in the TCGA cohort. **B** qRT-PCR analysis of CEP55 mRNA. **C** Differential protein expression of 4 model genes in normal and RCC samples in the CPTAC cohort. **D** Kaplan–Meier survival analysis for CEP55 protein in the CPTAC cohorts

We examined the mRNA expression levels of Prol signature genes within RCC and non-cancerous samples by acquiring data from the TCGA cohort. We noticed that these Prol signature genes were significantly upregulated in RCC samples (all *P* < 0.001) as depicted in Fig. 8A. Among this group, CEP55 emerged as the most vital gene, as per the RSF evaluation (Fig. 4B). Further validation of CEP55 expression was achieved through qRT-PCR analysis and consultation of the CPTAC database. The qRT-PCR result demonstrated a substantial increase in CEP55 mRNA expression levels compared to normal tissues within the Changhai cohort (*P* < 0.001) (Fig. 8B). The CPTAC database further confirmed this, with CEP55 protein expression markedly elevated in RCC tissues in contrast to normal kidney tissues (*P* < 0.001) as shown in Fig. 8C. Additionally, an adverse correlation was observed between high CEP55 protein expression and OS in RCC patients as recorded in the CPTAC database (*P* < 0.05) (Fig. 8D). These findings underline that the anomalous expression of these genes, particularly CEP55, might contribute significantly to the processes of RCC tumorigenesis and progression.

## Discussion

In this study, we utilized scRNA-seq data derived from RCC and normal renal tissues to construct a comprehensive transcriptomics framework that facilitated the decomposition of cell types in bulk RNA-seq data collected from the TCGA cohort. We identified a proliferative cell type, termed ‘Prol,’ which exhibited significant proliferation and was distinctly associated with RCC, with its presence correlating to notably poorer survival outcomes. Analysis of Prol-specific marker genes, identified from our scRNA-seq data, revealed elevated expression linked more adverse survival outcomes in the TCGA cohort. Finally, we established a Prol signature by leveraging a novel AI network to quantify the prevalence of Prol cells and improve prognostic predictions, focusing on key genes.

Utilizing data from the TCGA, an invaluable resource for oncology research, we identified RCC-associated cell types and evaluated their clinical significance by integrating data from our scRNA-seq datasets. Past studies have drawn connections between several marker genes, which are unique to Prol cells, with inferior survival outcomes [37–39]. Notably, Prol cells express a combination of well-known RCC-associated genes and newly identified targets, including HIST1H4C, TUBA1B, and H2AFZ, which merit further exploration. Our methodology has successfully pinpointed over a hundred RCC-specific marker genes, offering significant insights into the intricate biology of RCC and paving the way for future studies.

Given the unfavorable prognosis of RCC, we investigated determinants influencing patient survival and discovered the potential of Prol cells as valuable indicators for outcome prediction. Currently, prognosis prediction for RCC relies heavily on tumor stage and metastasis [40]. Marker genes of Prol cells may herald a new era of prognostic technologies based on gene expression. With the advent of RNA sequencing, clinical laboratories can now identify gene expression patterns that are indicative of prognosis [41]. We developed a unique AI network to develop a Prol signature drawing on the expression profiles of Prol cells marker genes. This network integrated 11 algorithms from the realms of traditional regression, machine learning, and deep learning, and surpassed the predictive performance of previous machine learning frameworks [18]. Notably, the optimal combination was VSOLassoBag and RSF, a pairing overlooked in previous studies [18]. Employing the AI network for feature pruning, model optimization, and generalization efficacy enhancement is beneficial. ROC and C-index analysis revealed the Prol signature to predict outcomes in all four cohorts effectively, indicating its potential for clinical application. Notably, none of the treatments, including sunitinib, everolimus, or the combination of nivolumab and atezolizumab with bevacizumab in multiple cohorts, improved the prognosis of patients with elevated Prol signature scores, indicating a need for the development of novel therapeutic agents. This suggests that current pharmaceutical interventions may be indifferent to Prol cells. Furthermore, the Prol signature showed pan-cancer prognostic ability, implying that similar cell types may exist in these tumors and deserve further study.

Despite the progress made in understanding the clinical significance of Prol cells, this study has limitations. Our study cohorts exhibit heterogeneity due to the use of various sequencing and microarray platforms. Although we applied standard normal transformations to address this disparity, its impact on the results remains a consideration. Furthermore, validation of the Prol signature’s clinical utility requires larger, independent cohorts. This will not only confirm its prognostic value but also allow us to investigate its potential for guiding personalized treatment strategies. Future research should prioritize isolating this tumor subcluster to investigate its biological functions. This deeper understanding of the molecular mechanisms driving tumor development could uncover novel therapeutic targets.

## Conclusion

In summary, by making use of both the scRNA-seq and bulk RNA-seq data, we succeeded in identifying a previously unexplored cell type, characterized by its cell cycle phase, the activity of transcription factors, and its prognostic significance. This study paves the way to novel understandings of the molecular and cellular mechanisms in RCC, simultaneously driving the development of novel biomarkers and therapeutics.

## Data Availability

The data used to support the findings of this study are available from the corresponding author on reasonable request.
